# Children and Adolescents Psychological Distress Scale During COVID-19 Pandemic: Validation of a Psychometric Instrument (CONFEADO Study)

**DOI:** 10.3389/fpsyt.2022.843104

**Published:** 2022-08-08

**Authors:** Carla De Stefano, Isaura Laurent, Véronique-Carelle Kaindje-Fondjo, Mégane Estevez, Enguerrand Habran, Bruno Falissard, Pascale Haag, Imane Khireddine, Fabien D'Hont, Thierry Baubet, Nicolas Oppenchaim, Stéphanie Vandentorren, Dalila Rezzoug

**Affiliations:** ^1^Université Paris 13 Sorbonne Paris Nord, Bobigny, France; ^2^AP-HP, Urgences - Samu 93, Hôpital Avicenne, Bobigny, France; ^3^AP-HP, Department of Child and Adolescent Psychiatry and General Psychiatry, Avicenne Hospital, Bobigny, France; ^4^Centre National de Ressources et de Résilience Lille-Paris (CN2R), Lille, France; ^5^Ecole Nationale de Statistique et Analyse de l'Information (ENSAI), Bruz, France; ^6^INSERM UMR 1219, Population Health, Phare Team, University of Bordeaux, Bordeaux, France; ^7^Fonds FHF Recherche et Innovation, Paris, France; ^8^Centre de Recherche en épidemiologie et santé des populations (CESP), Université Paris Saclay, Villejuif, France; ^9^Laboratoire BONHEURS, Ecole des Hautes Etudes en Sciences Sociales (EHESS), LabSchool Network, Paris, France; ^10^Santé Publique France, Scientific and International Direction, Saint-Maurice, France; ^11^Centre Hospitalier Universitaire de Lille Inserm, University Lille, LilNCog-Lille Neuroscience and Cognition, Lille, France; ^12^Université de Tours, UMR CITERES, Allée Ferdinand de Lesseps, Tours, France

**Keywords:** children, adolescents, mental health, COVID-19, distress, validation, psychometric, scale

## Abstract

**Aim and Object Purpose of the Study:**

In March 2020, the WHO declared a pandemic (COVID-19) due to the SARS-CoV-2 virus. In France, school closures and lockdowns were implemented. In this unprecedented context for French adolescents and children, the CONFEADO study surveyed children aged 9 to 18 years to assess their mental health, psychological distress, and resilience during and after the lockdown in relation to their living and housing conditions. To assess psychological distress, a psychometric tool (Children and Adolescent Psychological Distress Scale-CAPDS-10) was specifically designed for the research. This article presents the psychometric validity of the CAPDS-10.

**Methods:**

This cross-sectional study collected data from June 9 to September 14, 2020, from children and adolescents (9 to 18 years of age) via an online questionnaire after sending it to a large network of partners. Psychological distress, resilience, and trait anxiety were assessed using the CAPDS-10, the Child and Youth Resilience Measure (CYRM), and the State-Trait Anxiety Inventory for Children (STAIC). The CAPDS-10 measured perceived psychological distress in the most recent 2 weeks (primary endpoint). The predictive power of the CAPDS-10 was determined by statistical analysis. We proceeded to a confirmatory factor analysis to validate the scale at a clinical level. We carried out a psychometric validation with a step to verify the uni-dimensionality of the scale (PCA analysis) and the calculation of convergent and divergent validity, correlation coefficient between items and subscales, Cronbach's alpha for reliability, determination of a cut-off score for the AUROC index.

**Results:**

Three thousand and forty eight children and adolescents completed the CAPDS-10. Analysis confirmed a three-factor model (anxiety, depression, and aggressive behavior) (RMSEA = 0.072 [0.067; 0.077], CFI = 0.954), with a correlation coefficient between items >0.4. PCA analysis concluded that the scale is unidimensional. Reliability was satisfactory with Cronbach's alpha coefficients >0.7 (0.86). In addition, prediction was good with an AUROC index equal to 0.73 and a threshold score for severe distress greater than or equal to 19.

**Conclusion:**

The CAPDS-10 measures psychological distress over the most recent 2-week period with good psychometric qualities. It could be used in crisis or prevention contexts in the general population or in clinical settings.

## Introduction

Since early 2020, the whole world has been faced with a health crisis, resulting from the spread of the SARS-CoV-2 virus and COVID-19 (1). To contain the pandemic, the lockdown strategy was implemented by most countries around the world (2, 3), albeit without controlling all the consequences, in particular the psychological, psychiatric and social repercussions (4).

Early studies on the psychological impact of the lockdown showed a high prevalence of distress symptoms and psychological disorders (5–8). In groups of children and adolescents, initial findings highlighted an upswing in depressive and anxiety symtomatology (9–13), sleep and appetite disorders (14), anguish and worry related to disease (15), and behavioral disorders (16).

Most studies took the presence of symptoms of anxiety and depression as criteria for psychological distress. Other studies focused on the quality of life and sleep, on substance use (alcohol and tobacco) and on difficulties regulating emotions, with an impact on the children's and adolescents' relationships, emotions and behavior (17–19). All of this research indicates a wide range in the expression of psychological distress, but without being able characterize its severity.

We hypothesize that the COVID 19 pandemic, in connection with an emerging disease associated with the confinement of the population, is likely to cause psychological distress in children and adolescents in certain living conditions and context. The disease itself and the epidemic generate many uncertainties and anxieties (ex. variety of symptomatic expression with a deleterious evolutions, increasing number of deaths and hospitalizations in intensive care, scary media communication, overwhelmed health care system, risk of transmitting the disease to the most fragile relative). Moreover, the rupture of educational continuity and social life can lead to a loss of life habits of children and adolescents. In this double health and social constraint, anxious and depressive symptoms can appear, particularly in those who are deprived of their relationships (school, peer group). In addition, anxiety and depressive affects may be expressed in children and adolescents through somatic complaints (fatigue, physical pain, sleep). Moreover, the emotional context lead to dysfunctional manifestations in the relationships (opposition, irritability, more aggressiveness). These manifestations being exacerbated when there is no longer a third space such as school and social life to alleviate the weight of the reality of daily life within the families.

No self-report psychological distress screening tools evaluating children and adolescents aged 9–18 were currently available in French. K6 (French version available) and K10 (English version only) scales are used for screening distress but only in adolescents and adultes (20–22). In addition, although the KINDL scale (quality of life assessment) is available in French and at CONFEADO target ages, it does not meet our hypothesis (23). In fact, we were already exploring individual and relational resilience via the CYRM. We were aiming for a short, easy-to-complete scale that could be used in a variety of contexts (general or clinical population).

In France, school closures started on March 13, 2020, the general lockdown of the population began on March 17, 2020 and ended on May 10, 2020. The CONFEADO study focused on the emotional state and mental health of children and adolescents during and after the first lockdown. Within this framework, we produced a psychometric scale dealing with psychological distress, which is, from our viewpoint, clinically adapted to the experience of the crisis situation. The different registers as the anxiety, the depression, the somatic symptoms, the aggressiveness in relationships were integrated into our psychometric tool.

This article presents the psychometric validity of the tool as per psychological distress and the rules for interpreting the scale scores.

## Methods

### Development of the Scale

We followed several steps to develop the CAPDS-10. First, a review of the literature was conducted to find a scale translated into French that measures psychological distress in children and adolescents aged 9 years and older.

Relevant and possible items were pooled by the first two authors of the study (CDS and DL). After categorizing into internalizing and externalizing disorders, we selected sub-dimensions (depression, anxiety, aggressive behavior, relational difficulties, somatic pain/complaints). For each dimension we constructed items (20 items in total). After eliminating redundant items, another evaluation was carried out by the other authors of the article (SV, NO, TB) and by another child psychiatrist. Ten items were retained after having obtained a consensus in the group and a pilot phase started, in April 2020, with a group of children and adolescents who volunteered to assess the correct understanding of the items. A visual analog scale, ranging from 0 (not at all understandable) to 5 (completely understandable) was proposed. Eleven children participated in this pilot phase. The results show that the comprehension of our questions was rather good (mean age = 11.4; standard deviation = 2.6; mean global score = 4.5). After filling in the questionnaire, a mini interview was carried out by the first two authors in order to find out the children's suggestions for improving the tool.

### Participants and Procedure

The study population includes parents and their children, aged 9 to 18. The age range chosen allowed us to focus on childhood, preadolescence and adolescence periods. The inclusion criteria were as follows: children and adolescents aged from 9 to 18 years old, capable of giving their informed consent. Exclusion criteria were as follows: children under 9 or over 18 years old. The study took place from June 9, 2020 to September 14, 2020. At the start of the study, the lockdown had already begun, gradually and heterogeneously implemented throughout France. In fact, some children had already returned to school while others were still staying at home. This survey was authorized by a French research ethics committee, the *Comité de Protection des Personnes Ile de France VIII*, before its initiation (N°2020-A01342.37). Information was provided to all participants before their enrollment. The survey was anonymous. No compensation was offered. The link to the questionnaire was sent to families by various institutions or associations, such as the FCPE (Parent Association) and the UNAF (National Union of Family Associations), through partners of the University Sorbonne Paris-Nord, Santé Publique France (Public Health France - SPF), the National Observatory of Child Protection (ONPE) and the Paris Hospitals Public Assistance communication network (AP-HP). The wide scope of associations and institutions involved made it possible to reach a diverse group of children and adolescents in France. In addition, the link was transmitted via social media (e.g., Twitter, Facebook). For children in the care of child welfare services (ASE), the questionnaire was available on paper through childcare workers willing to recruit participants for the study.

### Measures

#### Demographic Informations

The questionnaire was made anonymous, standardized, and developed from a multidisciplinary perspective (public health, psychology, psychiatry and sociology). It had a section for parents or adult caregivers, followed by a section for youth (children/adolescents). A system using vocal synthesis capable of reading the questions and answers was provided in case of illiteracy. The questionnaire completed by parents collected socio-demographic data (gender, age, municipality of residence, employment status, occupation, diploma, nationality, perception of the financial status of the household).

The questionnaire completed by children collected:

- socio-demographic data (e.g. gender, age, family bilingualism…)
- data regarding the child's/adolescent's general physical condition and emotional state (e.g. sleep, appetite, emotions upon waking-up and at bedtime) for discriminant validity.

#### State-Trait Anxiety Inventory for Children (STAIC)

Screening trait anxiety in children aged 9 to 18 in the event that the child experienced stressful events unrelated to Covid-19. For that screening, we used the *State-Trait Anxiety Inventory for Children (STAIC)* (24). It is a self-report tool completed by the children themselves and includes 10 items rated from 1 to 3 points, leading to a total score between 20 and 60 points. A high score indicates a child with trait-anxiety characteristics. The STAIC was used for the concurrent validity of the CAPDS-10.

#### Child and Youth Resilience Measure

However, to evaluate resilience, we used the score from the Child and Youth Resilience Measure (CYRM-R) (25). It is a self-report tool completed by the children themselves, which includes 17 items each rated from 1 to 5 points, leading to a total score from 17 to 85 points. A high score indicates a child with characteristics of resilience. The CYRM-R was used for the concurrent validity of our instrument.

### Statistical Analysis

Analyses for the CAPDS-10 validity were done using STATA 12.1 and R 4.3.0, with the valid scale module. The initial tool construct was divided into four dimensions: Depression (Items 1–3), Anxiety (Items 4–6), Somatic Complaints (Item 7), and Aggressive Behavior (Items 8–10).

#### CAPDS-10 Validity

##### Confirmatory Analysis

The confirmatory analysis was carried out to ensure the clinical validity of the tool in terms of the items chosen to assess depressive, anxiety and aggressiveness-related symptoms.

In order to confirm the correlations between the batches of hypothesized items and the latent variables associated with the initial dimensions, a Confirmatory Factor Analysis (CFA) was used. The parameters estimated were intercepts, factor loading, error variance, and the parameters of the latent variables. Maximum likelihood was the estimation procedure. Model adequacy was evaluated by a chi-squared test, but that test is more likely to be significant when the number of individuals is high (in this case, *N* = 3,148); it was therefore not the main indicator to be taken into account. The construct was actually confirmed through several indices: the root mean square error of approximation (RMSEA), Comparative Fit Index (CFI) and Tucker-Lewis Index (TLI). An RMSEA <0.08 is considered to represent a good fit, and the CFI reflects a good fit if it is >0.9. Factor loadings should be at least >0.4, and values >0.7 demonstrate very good correlations between the items and the latent variables of the subscales [26, 27].

##### Uni-Dimensionality Check

Since the CAPDS-10 scale is built-up from different items, the underlying assumption is that the construct is dominantly uni-dimensional. In order to check that assumption, we conducted a principal component analysis (PCA). PCA is a statistical technique that allows to summarize the information content in some variables by means of a smaller set of summary indices called factors or dimensions. Analyzing the fraction of total variance captured by a single factor, we will be able to determinate if the scale can be considered uni-dimensional.

##### Convergent Validity

Convergent validity was evaluated by studying the matrix of correlations between the items and the scale. The correlation coefficient between each item and the scale should be >0.4 to have convergent validity.

##### Reliability

The reliability of the scale was evaluated using Cronbach's alpha. The measure is considered precise enough if the said coefficient is >0.7.

##### Discriminant Validity

The hypothesis that the score increases with distress was confirmed by an analysis of its association with other variables present in the CONFEADO study questionnaire. For that analysis, one-way ANOVA tests as well as pairwise-tests (with Bonferroni correction) were conducted. The variables included were: “I feel sad in the morning,” “I feel worried in the morning,” “I feel happy in the morning,” and their three equivalents for the evening. Those variables are related to the child's emotions all had the following response modes: “No,” “Yes, a little,” “Yes, very.”

##### Concurrent Validity

External validity was done using CYRM-R and STAIC scales. The correlation coefficients between those scales and the CAPDS-10 scale were calculated.

##### Determining a Distress Threshold

This distress screening tool requires determining a threshold score, called the “cut-off” score, starting at which a child can be declared to have a high likelihood of being in severe distress. An initial indication of this threshold can be given by the 95% quantile of the distress score. However, the threshold was truly determined by maximizing accuracy with the creation of a “real distress” variable.

That “real distress” variable was created using six other variables included in the rest of the CONFEADO survey questionnaire, which were: “I feel sad in the morning,” “I feel happy in the morning,” “I am afraid in the morning” and their equivalents for the child's feelings in the evening. The response modes were “No,” “Yes, a little” or “Yes, very.” Those variables were combined to limit their number to three binary variables: “I feel sad overall,” “I feel happy overall,” and “I'm afraid overall.” Children were considered to be sad overall if they had indicated that they felt “a little” or “very” sad in the morning and in the evening, and likewise for being afraid. Children were considered not to be happy overall if they had indicated that they didn't feel happy at all or just “a little” in the morning and in the evening.

The combination of these three binary variables resulted in a “real” distress mini-score between 0 and 3, which itself led to the creation of a “real” distress binary variable. That variable was the basis for determining the distress score threshold. Accuracy was calculated for all threshold values between 0 and 30:


$$\begin{array}{l} Accuracy\left(threshold\right)=\frac{TP+TN}{TP+TN+FP+FN}=1\\ -error\;rate\left(threshold\right) \end{array}$$

Where TP is the True Positives rate, TN the True Negatives rate, FP the le False Positives rate, and FN the False Negatives rate.

The goodness of fit of prediction was established by calculating the AUROC (Area Under the Receiver Operating Characteristic) curve. An AUROC >0.7 is a sign of goodness of fit.

A severe distress threshold on this tool was therefore detected by maximizing the accuracy while minimizing the threshold to keep from missing any cases of distress.

## Results

### Sample Characteristics and Population Selection

**Figure 1:**
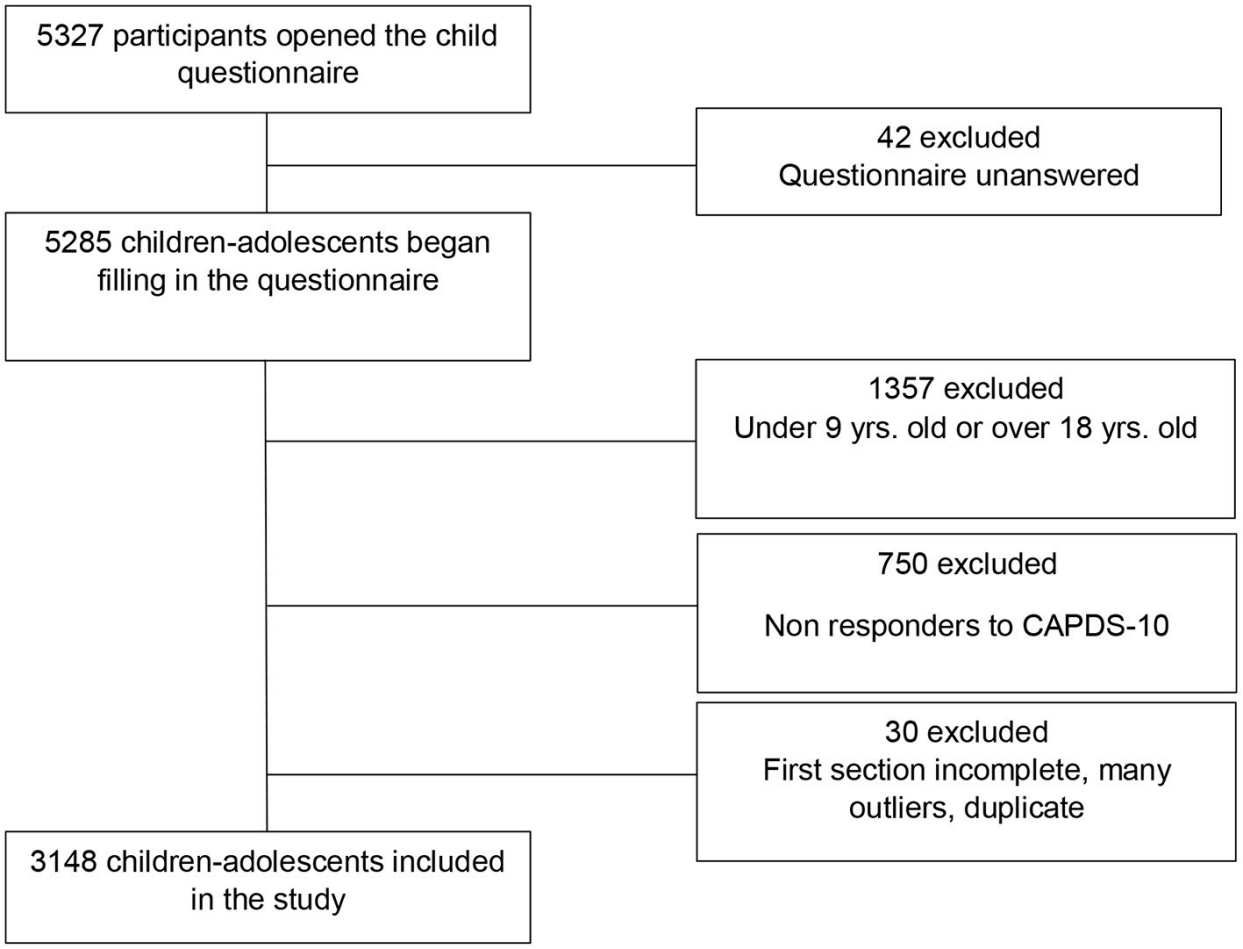
Flow chart CONFEADO study, France, 2020.

A total of 5,327 participants gave their consent and opened the questionnaire (Figure 1). Three thousand and forty eight children and adolescents were included in the study (Table 1).

**Table 1:** Sample characteristics (*N* = 3,148).

**Variables**	**9-11 years old (*N* = 426) *N*(%)**	**12-14 years old (*N* = 496)** ***N*(%)**	**15-18 years old (*N* = 2,226) *N*(%)**
**Sex (N)**	**426**	**496**	**2,226**
Girl	208 (48.8)	267 (53.8)	1,731 (77.8)
Boy	218 (51.2)	229 (46.2)	495 (22.2)
**Joint custody (N)**	**426**	**496**	**2,226**
No	402 (94.4)	458 (92.3)	2,045 (91.9)
Yes	24 (5.6)	38 (7.7)	181 (8.1)
**Child protection care (N)**	**426**	**496**	**2,226**
No	414 (97.2)	484 (97.6)	2,205 (99.1)
Yes	12 (2.8)	12 (2.4)	21 (0.9)
**History of mental disorders (N)**	**424**	**493**	**2,221**
No	345 (81.4)	413 (83.8)	1,649 (74.2)
Yes	79 (18.6)	80 (16.2)	572 (25.8)
**Psychological distress (CAPDS-10) (N)**	**426**	**496**	**2,226**
No or mild distress	323 (75.8)	366 (73.8)	1,240 (55.7)
Moderate distress	90 (21.1)	101 (20.4)	788 (35.4)
Severe distress	13 (3.1)	29 (5.8)	198 (8.9)
**Nationality (N)**	**424**	**493**	**2,221**
Two French parents	362 (85.4)	432 (87.6)	1,702 (76.6)
A foreign parent	46 (10.8)	46 (9.3)	322 (14.5)
Two foreign parents	16 (3.8)	15 (3.0)	197 (8.9)
**Parental social support (N)**	**424**	**493**	**2,221**
Yes	370 (87.3)	420 (85.2)	1,923 (86.6)
No	54 (12.7)	73 (14.8)	298 (13.4)
**Family structure (N)**	**409**	**483**	**2,108**
Two parent or blended families	352 (86.1)	372 (77.0)	1,563 (74.1)
Single parent	57 (13.9)	111 (23.0)	545 (25.9)
**Parents' occupational category (N)**	**397**	**466**	**1,887**
Farmers	2 (0.5)	2 (0.4)	23 (1.2)
Artisans	8 (2.0)	8 (1.7)	90 (4.8)
Executives	128 (32.2)	148 (31.8)	249 (13.2)
Intermediate occupations	144 (36.3)	164 (35.2)	443 (23.5)
Employees	86 (21.7)	112 (24.1)	674 (35.7)
Laborers	8 (2.0)	10 (2.1)	167 (8.8)
Retired or inactive	21 (5.3)	22 (4.7)	241 (12.8)
**Educational level (N)**	**424**	**493**	**2,221**
No diploma	21 (5.0)	37 (7.5)	377 (17.0)
High school diploma	87 (20.5)	102 (20.7)	917 (41.3)
Bachelor's degree	133 (31.4)	157 (31.9)	505 (22.7)
Master's degree	141 (33.2)	153 (31.0)	369 (16.6)
PH.D	42 (9.9)	44 (8.9)	53 (2.4)

*CONFEADO study, France, 2020. The meaning of the bold values is N*.

### CAPDS-10 Validity

The initial 4-dimension construct was questioned by the convergent and discriminant validity, and the impossibility to calculate Cronbach's alpha for the dimension “Somatic Complaints” which included only Item 7 (“I have felt physical pain, I have felt tired or I have had trouble sleeping”). Studying the matrix of the correlations between the items and subscales, as well as doing a principal component analysis (PCA) on the items, made it possible to assign that item to the “Depression” dimension. As such, the entire validity presented below was done on the tool divided into three subscales: Anxiety (three items), Depression (four items) and Aggressive Behavior (four items).

#### Confirmatory Analysis

The confirmatory analysis results showed that the three-factor model correctly fit the data observed. The factor loadings were all indeed very high, except for the items “have been restless or had trouble sitting still” and “not felt like doing things,” for which the values remained correct (>0.4). Moreover, the goodness of fit was very good (RMSEA = 0.072 [0.067; 0.077], CFI = 0.954). The chi-squared test confirming the model adequacy was also significant.

#### Uni-Dimensionality Check

**Figure 2:**
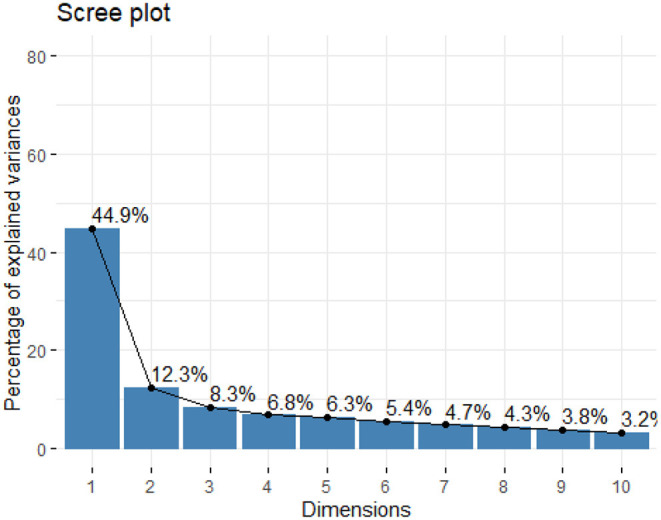
Graphic of uni-dimensionality test. CONFEADO study, France, 2020.

The PCA analysis produced the following graph (Figure 2). As one can see, the first-dimension concentrates about 45% of the information contained in the items while both the first and second dimensions concentrate 57%. We consider that this additional gain from the second dimension is negligible and keep only one dimension since 45% of variance is correct. We therefore conclude that the scale is uni-dimensional.

#### Convergent Validity

All 10 items had a correlation coefficient >0.4 with the scale (Table 2).

**Table 2:** Matrix of correlations between items and the scale.

**Items**	**Correlations**
1. I have felt angry, stressed out or worried	0.76
2. I haven't managed to overcome my stress or to deal with it	0.70
3. I have been restless or had trouble sitting still	0.59
4. I haven't felt like doing things or enjoyed doing things	0.60
5. I have felt discouraged or sad or unhappy	0.78
6. I have felt sluggish or I have felt a lack of energy	0.67
7. I have felt physical pain, I have felt tired or I have had trouble sleeping	0.70
8. I have disobeyed or I have opposed my parents	0.56
9. I have felt irritable or unpleasant or I have lost my temper	0.73
10. I have argued, I have had a fight, I have provoked other people	0.56

#### Reliability

The Cronbach's alpha value is 0.86 (>0.7) reflecting a good level of internal consistency of the scale.

#### Discriminant Validity

Table 3 presents the means and standard deviations of the CAPDS-10 score for all modes of the six emotion variables and the results of anova tests. For all those variables, the mean value of the score is higher when the child is very or little sad, worried or unhappy rather than happy or not worried at all. The anova tests revealed that for each of those emotion variables, the mean value of the score for at least one group is significantly different from the mean value of the score for the other groups (*p* <2.2e-16).

**Table 3:** Means of scale's scores for each mode of emotion variables.

	***N*(%)**	**Mean**	**Std deviation**	***P*-value**
**Feeling sad in the morning**				<2.2e-16 ^***^
No	2,015 (64.3%)	6.3	4.6	
little	920 (29.3%)	12	5.6	
Yes, very	199 (6.4%)	18	6.2	
**Feeling worried in the morning**				<2.2e-16 ^***^
No	1,796 (57.3%)	6.4	4.9	
little	1,026 (32.8%)	10	5.3	
Yes, very	311 (9.9%)	16	6.3	
**Feeling happy in the morning**				<2.2e-16 ^***^
No	1,057 (33.8%)	11	6.8	
little	1,520 (48.6%)	8.3	5.5	
Yes, very	549 (17.6%)	5.7	4.3	
**Feeling sad in the evening**				<2.2e-16 ^***^
No	2,088 (66.9%)	6.5	4.8	
little	702 (22.5%)	12	5.6	
Yes, very	332 (10.6%)	16	6	
**Feeling worried in the evening**				<2.2e-16 ^***^
No	1,833 (58.8%)	6.3	4.9	
little	970 (31.1%)	11	5.4	
Yes, very	314 (10.1%)	16	6	
**Feeling happy in the evening**				<2.2e-16 ^***^
No	1,485 (47.7%)	10	6.5	
little	1,240 (39.8%)	7.8	5.3	
Yes, very	391 (12.5%)	5.6	4.6	

*** *p-value < 0.05*.

In addition, we performed a pairwise *t*-test using Bonferroni's correction that confirmed significant difference between all pairs of groups of every single emotion variables.

#### Concurrent Validity

The correlation coefficients between the resilience and trait-anxiety scores, and the CAPDS-10 score are shown in Table 4. As one can see, psychological distress measured by the CAPDS scale is negatively and weakly related to the resilience while positively and strongly related to the anxiety.

**Table 4:** Correlation coefficients between the CAPDS-10 tool and the resilience and trait-anxiety scores.

	**CYRM-R Resilience**	**STAIC State-Trait Anxiety**
**CAPDS total Score**	−0.354	0.715

#### Determining a Cutoff Score

The “real” distress variable created through six emotion variables present in the rest of the questionnaire established that 545 (17%) children were in a state of distress, based on the emotions of sadness, fear and happiness.

**Figure 3:**
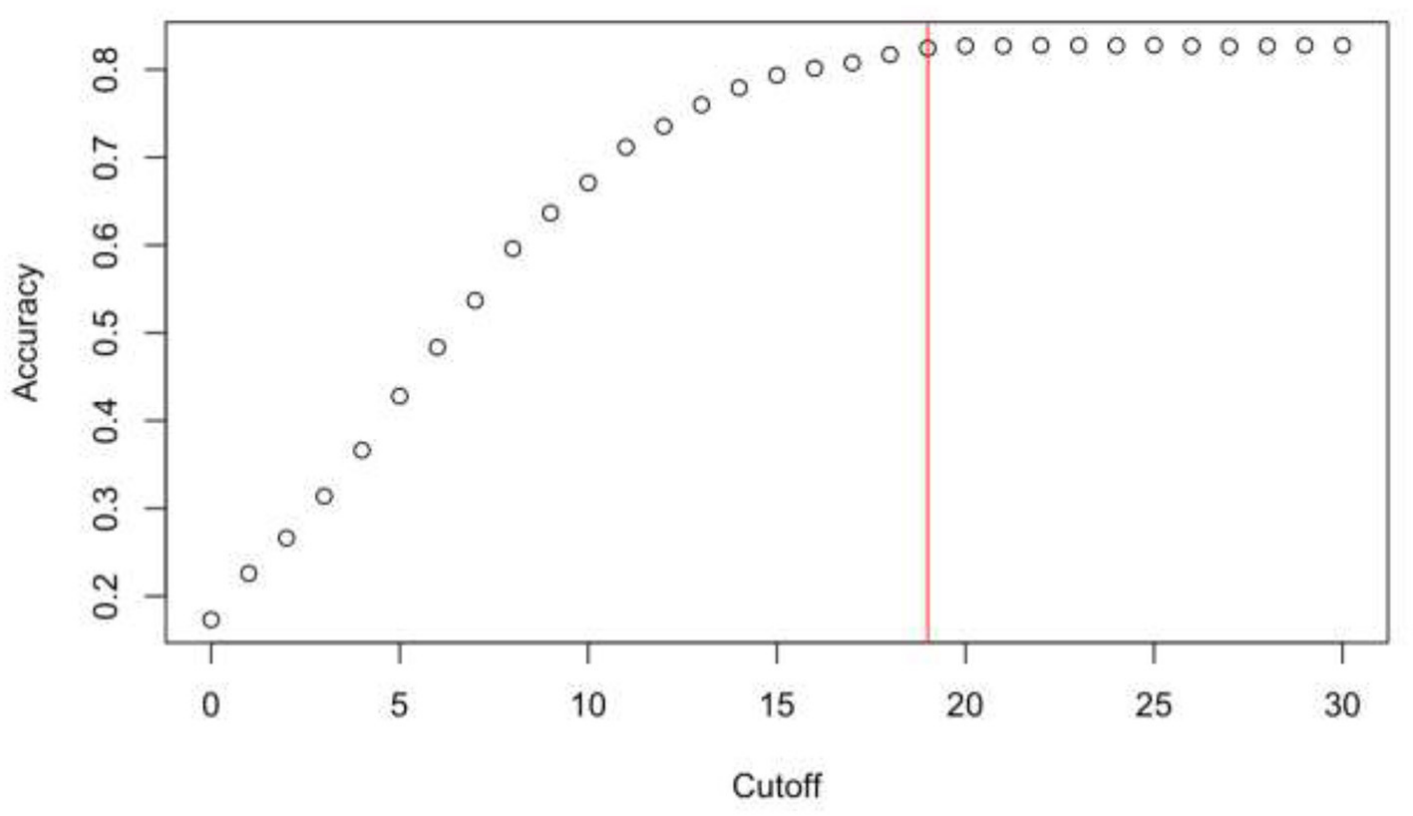
Accuracy as a function of threshold values.

Figure 3 shows the values of prediction accuracy as a function of each threshold on the distress variable from the CAPDS-10 tool, with the constructed variable of real distress as the base.

With the goal being to maximize accuracy while minimizing the threshold, it was established that a child is in a state of severe distress if s/he has a score ≥19. The AUROC index is equal to 0.73, therefore prediction is accurate. In addition, a score from 0 to 9 indicates no or mild distress, while a score from 10 to 18 indicates moderate distress.

## Discussion

This study presented the development of a new scale, Children and Adolescent Psychological Distress Scale - 10 items (CAPDS-10). Results showed that the CAPDS-10 has a stable unidimensional structure and robust psychometric properties. In addition, the overall score of the summed items can indicate the severity of psychological distress in children and adolescents. The internal consistency of the CAPDS-10 items was satisfactory with a Cronbach's alpha = 0.86. Based on the clinical distinction between depression, anxiety, aggressiveness and somatic complaints, a four-factor model was examined. Confirmatory analysis showed a three-factor model, aggregating somatic complaints to the depression factor.

In addition, some of the items proposed for the CAPDS-10 may be common to an anxiety and depression clinic such as irritability, or somatic symptoms. This is particularly true in children who express their suffering through aggressive behavior in relationships (externalized symptoms) due to a less mature emotional regulation capacity. Thus, treating psychological distress as a transnosographic dimension seems relevant. Given the original purpose of the CAPDS-10 to measure psychological distress and given the unidimensionality of the scale, we retained the simpler procedure of a global score for the measurement of psychological distress.

Concerning the construct validity of our tool, our results show a high and positive correlation of the CAPDS-10 with the STAIC scale and with the self-reported emotional experience of children and adolescents collected in the CONFEADO study questionnaire. Moreover, the CAPDS-10 is weakly and negatively correlated with the CYRM (−0.35), which means that the higher the resilience score, the lower the distress score. From a clinical point of view, resilience does not correspond to an absence of psychological distress but rather to a protective factor in interaction with other individual and environmental aspects of the child.

The clinical aim of the CAPDS-10 was to screen primarily children and adolescents with high levels of psychological distress. Clinically, we assumed a threshold of 20, which corresponds to symptomatic expression more than half the time for each item. This constitutes a demanding threshold. The calculation of the threshold for severe distress was statistically calculated. The statistical threshold (cut off score equal to and >19) is very close to the clinical score, which means that the child is in a state of severe distress. The threshold maximizes the AUC that leads to reduce the false positive rate by conserving a good detection rate. Regarding to this threshold, the CAPSD-10 has its optimal usage range above 19 score.

Several limitations must be taken into account in our study. We performed a single measure during the first confinement, which does not allow for comparison of scores with a pre-Covid measure. Furthermore, we could not verify the temporal stability of our tool in a test-retest setting. In addition, no depression scale was used to complete the concurrent validity. The scale was validated on 3,148 participants, with the loss of 750 participants who did not complete.

In conclusion, the CAPDS-10 is the first French scale, validated in general population, to detect psychological distress in children and adolescents aged 9 to 18 years, in self-report. This scale has good psychometric properties and is very quick to complete. It can be easily used by health professionals in individual and collective crisis situations.

## Ethics Statement

The studies involving human participants were reviewed and approved by Comité de Protection des Personnes Ile de France VIII (N°2020-A01342.37). Written informed consent to participate in this study was provided by the participants' legal guardian/next of kin.

## Author Contributions

Critical revision of the manuscript for important intellectual content: CDS, DR, IL, V-CK-F, ME, SV, NO, PH, FD'H, TB, BF, and IK. Study supervision: BF, TB, and FD'H. Wrote the manuscript: CDS, DR, IL, and V-CK-F. Contributed reagents, materials, and analysis tools: PH, FD'H, BF, TB, and IK. Analyzed the data: IL, V-CK-F, ME, CDS, DR, SV, and NO. Conceived and designed the experiments: CDS, DR, NO, SV, and BF. All authors contributed to the article and approved the submitted version.

## Conflict of Interest

The authors declare that the research was conducted in the absence of any commercial or financial relationships that could be construed as a potential conflict of interest.

## Publisher's Note

All claims expressed in this article are solely those of the authors and do not necessarily represent those of their affiliated organizations, or those of the publisher, the editors and the reviewers. Any product that may be evaluated in this article, or claim that may be made by its manufacturer, is not guaranteed or endorsed by the publisher.
